# Safety and Pharmacokinetic Assessment of Oral Proglumide in Those with Hepatic Impairment

**DOI:** 10.3390/pharmaceutics14030627

**Published:** 2022-03-12

**Authors:** Christine C. Hsu, Sunil Bansal, Hong Cao, Coleman I. Smith, Aiwu Ruth He, Martha D. Gay, Yaoxiang Li, Amrita Cheema, Jill P. Smith

**Affiliations:** 1 Department of Transplant Surgery, MedStar Georgetown University Hospital, Washington, DC 20007, USA; cch87@georgetown.edu (C.C.H.); coleman.i.smith@gunet.georgetown.edu (C.I.S.); 2 Department of Oncology, Georgetown University, Washington, DC 20057, USA; sb1886@georgetown.edu (S.B.); arh29@georgetown.edu (A.R.H.); yl814@georgetown.edu (Y.L.); akc27@georgetown.edu (A.C.); 3 Department of Medicine, Georgetown University, Washington, DC 20007, USA; hongc878@gmail.com (H.C.); mdg111@georgetown.edu (M.D.G.)

**Keywords:** cholecystokinin receptor, cirrhosis, hepatocellular carcinoma, pharmacokinetics

## Abstract

Proglumide is an orally administered cholecystokinin receptor antagonist that was found to improve nonalcoholic steatohepatitis, reverse liver fibrosis, and decrease incidence of hepatocellular carcinoma (HCC) in animal models. The current investigation aimed to test the pharmacokinetics and safety of proglumide in subjects with hepatic impairment compared with healthy controls. In this translational study, subjects with confirmed cirrhosis, Child-Pugh stage A or B, or healthy controls were recruited for a single-dosing study. Baseline urine and blood samples were obtained before administration of proglumide and also collected after ingestion up to 24 h. Drug concentrations measured by mass spectroscopy revealed peak plasma concentrations (Cmax) of 7847, 9721, and 10,635 ng/mL at about 1 h (Tmax) for healthy controls, subjects with Child-Pugh A, and B cirrhosis, respectively. The serum elimination half time was 3 h. Maximum urine drug concentration (Cmax = ~411 µg/mL) was observed at 3 h, and urinary drug concentration declined at 5 h. There were no adverse events reported, and follow-up liver panels in cirrhosis subjects were unchanged or improved. This investigation demonstrated that proglumide is safe and has similar pharmacokinetic properties in subjects with cirrhosis as in healthy controls; therefore, it will be safe to test the efficacy of proglumide as a therapeutic agent in those subjects with cirrhosis or HCC.

## 1. Introduction

Today, the fastest-growing cause of cancer-related death is hepatocellular carcinoma (HCC) [1]. Infection with chronic hepatitis B or C virus is currently the dominant risk factor worldwide, accounting for more than 1.3 million deaths per year from HCC [2]. Although vaccination for hepatitis B and treatment of hepatitis C has helped to decrease the liver cancer incidence from viral etiologies, the new obesity pandemic associated with the rising prevalence of nonalcoholic steatohepatitis (NASH) has become a new risk factor for cirrhosis and HCC [3]. Additionally, treatment of hepatitis C can reduce HCC risk, but does not eliminate risk [4], and despite universal HBV vaccination, prevalence remained constant at 0.3% from 1999 to 2016 in the United States because of persistent immigration by individuals from endemic areas for chronic hepatitis B [5].

The greatest risk factor for developing HCC is cirrhosis [6], and 80–90% of HCCs arise in background of a cirrhotic liver [7]. Cirrhosis is an increasing cause of morbidity and mortality and results in 1.03 million deaths per year worldwide [8]—170,000 per year in Europe and 33,539 per year in the USA [9]. In a recent large meta-analysis of five studies that included 1495 patients, stage 1 fibrosis was associated with a liver-related mortality rate (MRR) of 1.4, and stage 4 fibrosis (or cirrhosis) had an MRR of 42.3 [10].

The components of a premalignant liver and the liver extracellular matrix (ECM) are now well recognized as important factors involved in the initiation of HCC. The hepatic stellate cell (HSC) [11,12] is an important component of the ECM and plays an essential role in fibrogenesis and hepatic inflammation. Hepatic fibrosis results from the activation of tissue myofibroblasts or HSCs that proliferate, migrate, and produce ECM components, such as type I collagen, and express cytokines and chemokines [13]. HSCs often respond to inflammatory stimuli [14], such as cytokines and chemokines from macrophages [15] and other immune cells, resulting in HSC activation and migration. Chronic inflammation with the release of these inflammatory mediators with the activation of hepatic stellate cells results in changes of the liver extracellular matrix (ECM), which predisposes to the development of HCC. In cirrhosis, chronic inflammation mediated by persistent cytokine and chemokine production is a central process in the development of dysplastic nodules and HCC [16].

Currently, there are no FDA-approved drugs for the treatment of liver fibrosis, yet strategies to reduce hepatic inflammation related to NASH fibrosis are under investigation. Antifibrotic strategies have been attempted with agents that inhibit the activation of HSCs, agents that block HSC function, anticytokine, or anti–growth factor therapies, and of course, with the elimination of the insulting agent (such as alcohol or hepatitis virus). Unfortunately, since many of these compounds are not specific to the fibroblasts, they have side effects, including possible deleterious effects on other organs. The HSCs also play an important role in the normal function of the liver and are thought to help with regeneration after injury and maintenance of cell function [17]. Therefore, agents that eliminate or injure the HSCs would be counterproductive.

The cholecystokinin-B receptor (CCK-BR) is not found in normal liver tissue (mouse or human) but becomes overexpressed in NASH and HCC [18]. In fact, in [19] 35% of the mice on a NASH-inducing diet developed dysplastic nodules or HCC after 18 weeks, while none of the mice treated with a CCK-BR antagonist, proglumide, developed HCC. Further examination of the mouse NASH livers in this study showed that treatment with the CCK receptor antagonist, proglumide, rendered the liver microenvironment less carcinogenic by decreasing fibrosis, inflammatory chemokines, and cytokines and reducing M2-polarized macrophages [18]. In fact, treatment of mice bearing HCC tumors with proglumide monotherapy resulted in a significant reduction in cancer growth compared with controls [18].

Due to the liver’s important role in the processing of xenobiotics, including drugs and toxic compounds, impaired hepatic function may alter this process, leading to drug-induced liver injury (DILI) or harmful side effects [20]. Subjects with cirrhosis are vulnerable to DILI or toxic reactions from medications, and therefore, understanding the pharmacokinetics and metabolism of drugs is essential in this population. The purpose of this investigation was to test the safety, pharmacokinetics, and excretion of proglumide in human subjects with cirrhosis compared with healthy controls.

## 2. Materials and Methods

### 2.1. Study Populations

This prospective open-labeled clinical trial undertaken at Georgetown University was an investigator-initiated translational research study based upon a study in mice that showed reversal of hepatic fibrosis and prevention of hepatocellular carcinoma [19]. The protocol and sample size were approved by the Food and Drug Administration (FDA) and the Georgetown University Institutional Review Board (IRB), and the trial was registered on www.clinicaltrials.gov (accessed on 20 February 2022) (NCT04814602). The study was designed to evaluate the effect of hepatic impairment on the pharmacokinetics (PK) of proglumide as related to hepatic function (e.g., Child-Pugh cirrhosis) compared with healthy controls. The principal objective of this study was to develop dosing recommendations so that patients and practitioners can alter the dose and dosing interval appropriately in the presence of hepatic disease, noting that subsequent careful titration and observation are critical in this vulnerable population. Written informed consent was obtained prior to screening subjects for enrollment. The study consisted of two groups of adults (>18 years of age). The first group included healthy controls with normal renal and hepatic function, with normal hepatic transaminases, chemistries, coagulation studies, and complete blood count. The comparator group was those with Child-Pugh stage A or B cirrhosis. Subjects with significant cardiac, pulmonary, or renal disease were not eligible, and those with HIV or untreated viral hepatitis were excluded. Subjects with hepatic transplant or Child-Pugh stage C were excluded from this investigation. Additional exclusion criteria included: subjects with active alcohol or drug abuse in the prior 6 months, current moderate alcohol consumption, evidence of active gallbladder disease or gallbladder dyskinesia, hypersensitivity or anaphylaxis to proglumide, and lactating or pregnant females. The diagnosis of cirrhosis was confirmed by one of the following: liver biopsy, FibroScan, FibroSure, MR elastography, or nodular liver on radiographic imaging including computerized tomography or magnetic resonance imaging (MRI).

### 2.2. Study Intervention

The study medication, proglumide, chemically is (*RS*)-*N*^2^-benzoyl-*N*,*N*-dipropyl-α-glutamine. It is water soluble with a molecular weight of 334.41 g/mol. The bulk drug was manufactured according to GMP standards and at >99% purity by A.M.S.A. S.p.A.—COSMA S.p.A., Milan, Italy, and compounded by Custom Prescriptions of Lancaster, PA, into vegan capsules containing 400 mg each. Prescriptions were labeled and dispensed by the investigational pharmacist.

### 2.3. Study Design 

**Figure 1:**
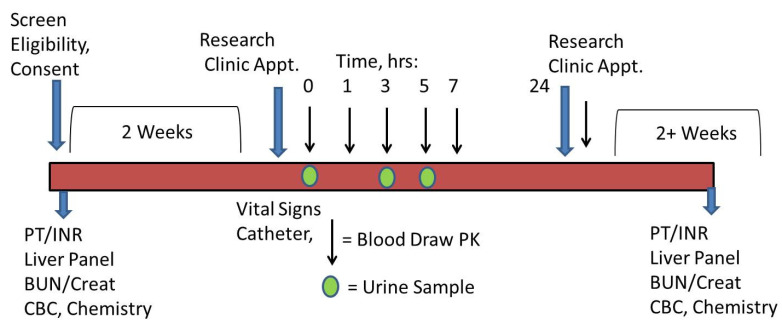
Study design. The schedule of events is shown. Black arrows show when blood samples were collected, and green circles when urine was collected. Proglumide 400 mg was administered by mouth. Visits denoted in blue arrows.

Male and female subjects were recruited from the outpatient clinics at MedStar Georgetown Transplant Institute and the Georgetown Lombardi Comprehensive Cancer Center (Figure 1). Healthy volunteers were recruited through local flyers at the university. After obtaining informed consent, subjects were screened with bloodwork, and a complete physical examination was performed for eligibility. Women of childbearing potential were required to undergo a urine pregnancy test. Eligible subjects were scheduled for a baseline visit in the Georgetown University Clinical Research Unit within 2 weeks of qualifying blood tests and exam.

Weight and height were obtained, and women of childbearing potential had another urine pregnancy test at baseline. A butterfly catheter was placed in the hand or arm from which a baseline (pretreatment) and subsequent blood samples (postingestion) were drawn. A baseline urine sample was collected. Proglumide 400 mg was administered orally, and blood samples were collected after ingestion at the following intervals ± 10 min: baseline, 1 h, 3 h, 5 h, 7 h, and 24 h (Figure 1). After 3 h and 5 h, subjects provided a urine sample. Vital signs (BP, pulse) were obtained and recorded at baseline and before each sample was drawn. Subjects were not fasting and were provided meals and water or beverages during the collection period. After the 7 h sample, subjects were discharged from the research unit and returned the following morning for the 24 h sample collection. A survey for adverse events was performed after 24 h and again 7–10 days after the treatment. Subjects were asked if they experienced any of the following new symptoms: abdominal pain, nausea and vomiting, loss of appetite, itching, chest pain, shortness of breath, trouble with urination, diarrhea or constipation, and headache or confusion. Follow-up serum chemistries and complete blood count were performed in cirrhosis subjects 7–14 days after proglumide ingestion.

### 2.4. Processing of Blood and Urine Samples

Samples were drawn in red top tubes, and serum was separated and aliquoted into 1.8 mL freezer vials, appropriately labeled with study subject number, date, and specimen collection time. Urine was also placed in a 1.8 mL freezer tube and labeled. Proglumide blood and urine samples were frozen at −80 °C, and samples batched and analyzed together for quality control in the Lombardi Core Metabolomic Laboratory by mass spectrometry.

### 2.5. Reagents and Chemicals

The following reagents were purchased: acetonitrile, isopropanol, and methanol from Optima Grade (Fisher Scientific, Waltham, MA, USA); high-purity formic acid (Thermo Scientific, Waltham, MA, USA); proglumide; and 4-nitrobenzoic acid (4-NBA) (Sigma-Aldrich, St. Louis, MI, USA).

### 2.6. Sample Preparation

The serum and urine samples were initially stored at −80 °C and thawed on ice (4 °C). The samples (serum 80 µL, urine 40 µL) were processed by the addition extraction buffer, that is, methanol (200 µL for serum, 360 µL for urine), containing 150 ng/mL of internal standard (4-NBA). The samples were vortexed for 30 s. The samples were then incubated on ice for 20 min, followed by incubation at −20 °C for 20 min. The samples were centrifuged at 13,000 rpm for 20 min at 4 °C. The supernatant was transferred to the mass spectroscopy sample vial for LC–MS analysis. A pooled quality control (QC) was generated by mixing 30 µL of each sample.

### 2.7. Preparation of Drug (Proglumide) Standards and Internal Standard Stock Solutions

Extraction buffer (Internal standard): A 1 mg/mL solution (Stock A) of 4-nitrobenzoic acid was prepared by dissolving 1 mg of 4-NBA in 1 mL of methanol and vortexed well for 30 s. An amount of 15 µL of Stock A was added to 100 mL of methanol to make the final nominal concentration of 4-NBA as 150 ng/mL. Proglumide (standard): A 1 mg/mL solution (Stock B) of proglumide was prepared by dissolving 1 mg of the drug in 1 mL of methanol and vortexed well for 30 s. Stock C (50 µg/mL) was prepared by making up 50 µL of Stock B up to 1 mL in methanol. To generate a calibration curve, Stock C was serially diluted in the extraction buffer to generate concentrations with control matrices in similar ratios as in actual test samples. Likewise, another set of standards in the similar concentration range was prepared using extraction buffer in blank solvent (methanol) for recovery estimation. Additionally, 3 QC samples were prepared in blank matrices and solvent using extraction buffer.

### 2.8. Liquid Chromatography–Mass Spectrometry (LC–MS) Methods

An MRM-based mass spectrometry method was developed for the measurement of the drug proglumide by UPLC–MS system. The samples were resolved on an Acquity UPLC BEH C18, 1.7 µm, 2.1 × 100 mm column online, with a triple quadrupole mass spectrometer (Xevo TQ-S, Waters Corporation, Milford, MI, USA). The liquid chromatography (LC) gradient method (Supplementary Materials, Table S1) started with 100% of mobile phase A (0.2% formic acid in water) that involved a gradient change from 0% B (0.2% formic acid in acetonitrile) to 80% phase B in 1.5 min after an initial lag phase of 1.5 min with the flow rate changing from 0.2 mL/min to 0.35 mL/min at 1.5 min and to 0.5 mL/mL at 3 min. The column was maintained at 40 °C, and injection volume was kept at 5 µL. An autosampler was maintained at 10 °C. The column eluent was introduced directly into the TQS mass spectrometer by electrospray operating in negative mode at a capillary voltage of 2.9 kV and a sampling cone voltage of 113 V. The desolvation gas flow was set to 1000 L/h, and the desolvation temperature was set to 500 °C. The cone gas flow was 150 L/h, and the source temperature was set to 150 °C. The sample cone voltage and collision energies were optimized for the analyte to obtain maximum ion intensity for parent and daughter ions using the “IntelliStart” feature of MassLynx software (Waters Corporation). The instrument parameters were optimized to gain maximum specificity and sensitivity of ionization for the parent and daughter ions. Signal intensities from the MRM Q1 > Q3 ion pairs for the drug proglumide (333.1 > 120.9) and 4-NBA (166 > 122, IS) were ranked to ensure selection of the most intense precursor and fragment ion pair for MRM-based quantitation.

For calibration curves, a series of concentration points (serum, 10 µg/mL to 1.0 ng/mL, and urine, 100 µg to 1.0 ng/mL) were used. The drug proglumide was detectable at as low as 2.5 ng/mL and 1.0 ng/mL in the matrices serum and urine, respectively. Similarly, the quantification limits for the serum and urine were in the range of 5 ng/mL to 10 µg/mL and 2.5 ng/mL to 100 µg/mL, respectively. The samples were prepared in duplicate (*n* = 2), and a sample queue was randomized prior to data acquisition. Standards were injected twice at the start and end of the batch to check whether there was any fall in drug response (due to degradation) during data acquisition. To nullify the matrix effects, calibration curve/QC samples were prepared by spiking standard drug in blank matrices (plasma and urine) in similar ratios (serum, 1:2.5, and urine, 1:9) as for the actual sample preparation. The linearity of drug response in calibration curve points was ascertained by including three quality control (QC) samples in the batch (serum, 60, 600 and 6000 ng/mL; urine, 400, 4000, and 40,000 ng/mL) at the start and end of the batch. Standard QCs (in extraction buffer and plasma) and pooled QC samples were injected periodically to monitor the consistency in the drug response at a particular drug concentration and monitor instrumental variance. Blanks (solvent alone) were injected on either side of test samples to assess sample-to-sample carryover. The details for the calibration curves for serum samples are shown in Supplementary Materials, Table S2. A sample chromatogram of eluted proglumide and internal standard (4-NBA) using UPLC-MRM-based mass spectrometric analysis of proglumide in serum is shown (Supplementary Materials, Figure S1).

The details for the calibration curves for urine samples are shown in Supplementary Table S3. A sample chromatogram of eluted proglumide and internal standard (4-NBA) by UPLC-MRM-based mass spectrometric analysis of proglumide in urine is shown (Supplementary Materials, Figure S2).

As a measure of assay reproducibility and drug stability, the data were acquired for three consecutive days, and intra-/interday variations were monitored. Data were processed using TargetLynx 4.1. The relative quantification values of analytes were determined by calculating the ratio of peak areas of transitions of the drug proglumide normalized to the peak area of the internal standard. For estimation of recovery, drug standards were prepared in extraction buffer, and control matrices and relative responses were measured.

### 2.9. Statistical Analysis

Means ± SEM for serum chemistries and blood counts were determined for controls and the two subcohorts of cirrhosis subjects. Analysis was evaluated with the assistance of GraphPad Prism 9 with ANOVA between groups considered significant with *p* < 0.05. Cmax (peak serum/urine concentrations), Tmax (time to reach Cmax), and T1/2 (elimination half time) were calculated for each subject and then averaged for each of the three separate groups. Differences between Cmax and Tmax were compared by two-way analysis of variance. Bioequivalence for Cmax and AUC between groups were estimated by Welch’s two-sample *t*-test without equal variance assumption using R (version 4.0.2).

## 3. Results

### 3.1. Patient Demographics and Baseline Laboratory Values

Eleven subjects enrolled in the study, including 3 African Americans, 2 Asians, and 6 Caucasians. The etiology of liver cirrhosis included the following: alcoholic cirrhosis (N = 2), NASH cirrhosis (N = 2), cryptogenic cirrhosis (N = 1), autoimmune overlap with primary sclerosing cholangitis (N = 1), and viral hepatitis B (N = 1). Two subjects were also undergoing therapy for HCC.

The demographics of the subjects are shown in Table 1. There was no statistical difference in the mean ages between the three groups. Body mass index (BMI) was greater in those with cirrhosis compared with healthy controls, but this difference did not reach significance. All subjects had normal renal function. Serum albumin values were 17% lower in those with Child-Pugh B cirrhosis compared with controls, but these values were not significant. MELD scores were increased in those with cirrhosis compared with healthy controls (*p* = 0.043). Prothrombin time was prolonged (*p* = 0.02), and total bilirubin levels were lower (*p* = 0.03) in subjects with cirrhosis compared with healthy controls.

### 3.2. Determination of Proglumide Levels by Mass Spectrometry

#### 3.2.1. Serum Proglumide Levels

**Figure 2:**
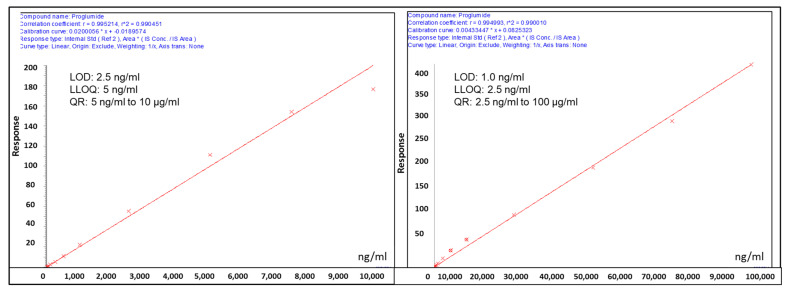
UPLC-MRM mass spectrometry analysis of proglumide: (**A**) Calibration curve for proglumide in serum over a range from 5 ng/mL to 10 µg/mL shows linearity. (**B**) Calibration curve for proglumide in urine over a range from 2.5 ng/mL to 100 µg/mL. (LOD = limit of detection; LLOQ = lower limit quantification, QR; quantification range). Calibration curve details have been included in the Supplementary Materials in Tables S2 and S3.

The serum proglumide calibration curve performed over a range of concentrations (10 µg/mL–5.0 ng/mL) demonstrates linearity (Figure 2A). Once maximum, the drug concentration was reduced to half in about 2.5 h (Cmax_1/2_). In general, the drug showed T_1/2_ of about 3.0 h. Drug response was stable over the duration of 3 days during data acquisition for most of the quantification range, and assay was reproducible and repeatable. This was ascertained by measurement of the drug response in the standard samples run at the start and end on each day. The limit of detection (LOD) for the drug was observed to be 2.5 ng/mL, and the limit of quantification as low as 5 ng/mL (LLOQ) with the response linearity maintained up to 10 μg/mL. Drug was quantifiable within the quantification range of 5 ng/mL to 10 μg/mL. Due to matrix effects, the drug responses varied from 80% to 105% from standards prepared in control plasma relative to when prepared in solvent blanks. Coefficient of variance (CVs) for Pooled QC and STD QCs (in matrix) remained well within limits (<15%) on all 3 days during data acquisition. CVs for intraday and interday when compared with day 1 were within 20%. As ascertained by blanks run on either side of sample sets, no sample-to-sample carryover was observed.

#### 3.2.2. Urine Proglumide Levels

Similarly, linearity is demonstrated with the proglumide urine calibration curve (Figure 2B) over a range of concentrations (5 ng/mL–100 µg/mL). Maximum drug concentration (Cmax = ~411 µg/mL) was observed at 3 h (Tmax). Subsequently, the drug concentration decreased until 5 h. The drug response was stable over the duration of 3 days across a broad quantification range. The assay repeatability and reproducibility was ascertained by measurement of the drug response in the standard samples run at the start and end on each day over 3 days. The limit of detection (LOD) for the drug was 1.0 ng/mL, and the limit of quantification as low as 2.5 ng/mL (LLOQ) with the response linearity maintained up to 100 μg/mL. Drug was quantifiable within the quantification range of 2.5 ng/mL to 100 μg/mL. Due to matrix effects, the drug responses varied from 50% to 85% (lower QR) and 30% to 40% (higher QR) for standards prepared in control urine relative to when prepared in solvent blanks. Pooled QCs and STD QCs (in matrix) showed coefficients of variance (CV) well within limits (<15%) on all 3 days during data acquisition, while for intra- and interday data, CVs were within 11%. No sample-to-sample carryover was observed as ascertained by blanks run on either side of each sample set.

### 3.3. Proglumide Pharmacokinetic Values in Human Subjects

**Figure 3:**
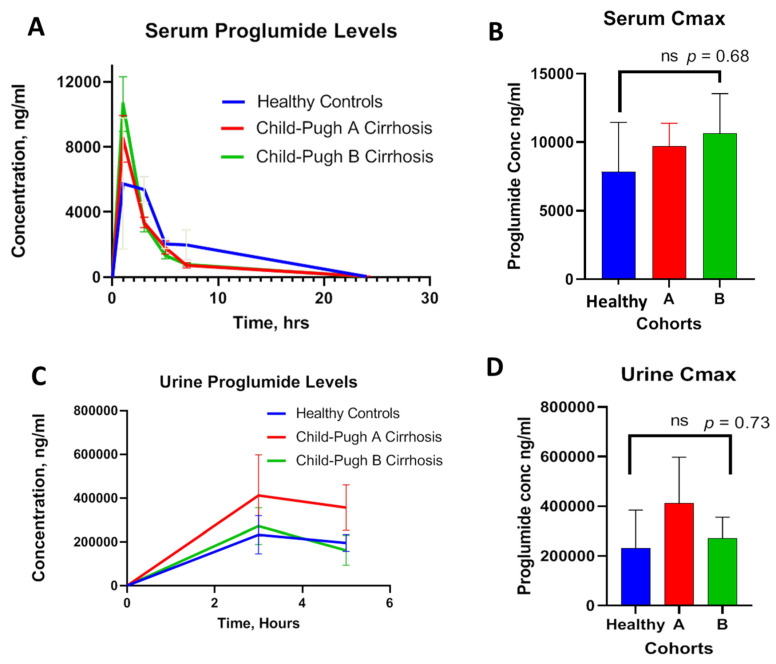
Measurements of proglumide levels in healthy controls and cirrhosis subjects. (**A**) Serum PK levels of proglumide over time in healthy controls compared with proglumide PK levels in subjects with Child-Pugh A or Child-Pugh B cirrhosis. (**B**) Peak serum concentration (Cmax) for each group shows no significant difference between the groups. (**C**) Proglumide urinary concentrations (clearance) over time are shown for healthy controls compared with proglumide PK levels in subjects with Child-Pugh A or Child-Pugh B cirrhosis. (**D**) Peak urinary concentration Cmax is shown for each group, and the values are not statistically different. Values are plotted as Means ± SEM.

The serum concentrations of proglumide at baseline and over time were plotted in the healthy controls and in Child-Pugh A and B cirrhotic subjects. Results revealed overlapping curves between the three cohorts with similar absorption and distribution over time without significant difference (Figure 3A). The mean peak serum concentrations (Cmax) were 7847, 9721, and 10,635 ng/mL for healthy controls, Child-Pugh A, and Child-Pugh B cirrhosis, respectively. There was no significance difference in Cmax between the three groups (Figure 3B). The time to reach Cmax (Tmax) was about 1 h in healthy controls and also 1 h in those with cirrhosis. Serum Cmax was reduced to half in about 2.5 h with an overall T_1/2_ of about 3.0 h. Proglumide plasma clearance expressed in terms of elimination rate constant (K_el_) were 0.22/h, 0.21/h, and 0.24/h for healthy controls, Child-Pugh A, and Child-Pugh B cirrhosis, respectively. Urinary clearance of proglumide was also measured over 5 h and compared between the groups. Mean urine proglumide levels over time for each group are plotted in Figure 3C. No significant difference in urinary Cmax values was appreciated (Figure 3D). The maximum urine drug concentration (Cmax = ~411 µg/mL) was observed at 3 h (Tmax), and the urinary drug concentration declined at 5 h, suggesting that urinary clearance of proglumide in cirrhosis subjects was comparable to healthy controls.

### 3.4. Average Bioequivalence for the Parallel Study

The method used to analyze the average bioequivalence in this investigation is suggested by the FDA “Guidance for Industry Statistical Approaches to Establishing Bioequivalence” (section VI. B.1.d). Statistical analysis for pharmacokinetic measures, including area under the curve (AUC) and peak concentration (Cmax), was based on the two one-sided tests procedure to determine whether the average values for the pharmacokinetic measures determined after administration of proglumide in healthy controls and those with cirrhosis were comparable. The average bioequivalence involves the calculation of a 90% confidence interval for the ratio of the averages (population geometric means) of the measures for the proglumide concentration in healthy controls compared with those with cirrhosis. According to FDA guidelines, to establish bioequivalence, the calculated confidence interval should fall within a bioequivalence limit, usually 80–125% for the ratio of the product averages. Results for statistical bioequivalence determined by Welch’s two-sample *t*-test for the healthy controls compared with those with Child-Pugh A or B cirrhosis are shown in Table 2. No significant difference was observed between the groups.

### 3.5. Safety Analysis

None of the healthy controls or cirrhosis subjects experienced any side effects related to ingestion of proglumide. One healthy control experienced bruising at the IV catheter site. One cirrhosis subject with hepatocellular cancer who had been experiencing severe leg pain noted that after ingesting proglumide, her pain resolved and she slept very well the night after taking proglumide.

Relevant pretreatment and post-treatment laboratory tests are shown in Table 3 for those with Child-Pugh A or B cirrhosis. No significant changes were identified in laboratory tests in regard to chemistries and complete blood counts. In fact, the tests related to the liver (transaminases, prothrombin time, and platelet counts) all improved slightly after ingestion of proglumide.

Another important feature of this study was that the administration of proglumide did not worsen hepatic function or raise hepatic transaminases in the follow-up phase, suggesting its safety in this population. Furthermore, there were no adverse events reported with this study including laboratory safety monitoring, vital sign monitoring, and patient reported side effects in those with cirrhosis.

CCK peptide is widely distributed throughout the brain, where it possesses properties of a neurotransmitter [21]. There are both CCK-A and CCK-B receptors in the nervous system located in the brain [22] and peripherally [23]. In this regard, proglumide was found to induce antinociception in mice [24]. Recent evidence suggests that the CCK peptide may interact with opioid receptors to alter pain [25]. Watkins et al. [26] observed that proglumide potentiates morphine analgesia following systemic, intrathecal, or intracerebral administration of these drugs. In Europe and Asia, proglumide has been used in combination with nonsteroidal anti-inflammatory agents (i.e., proglumetacin: proglumide and indomethacin) for pain management and analgesia [27]. In our current study, one subject with pain reported significant relief after ingestion of proglumide. This analgesic property may be beneficial in treating hepatic-impaired subjects with cirrhosis where opiates are contraindicated.

## 4. Discussion

In this clinical study, we found that the oral administration of the cholecystokinin receptor antagonist proglumide has similar pharmacokinetic properties in human subjects with Child-Pugh A and Child-Pugh B cirrhosis compared with healthy controls. Proglumide was rapidly absorbed with peak blood levels within approximately an hour after ingestion. The majority of proglumide was cleared from the circulation within 24 h and was excreted in the urine. Laboratory and clinical assessment of subjects revealed that proglumide did not cause any adverse events.

Proglumide is a derivative of glutamic acid and has antagonist properties at both the CCK-A receptor and CCK-BR [28]. Lorglumide, loxiglumide, and dexloxiglumide are other glutamic acid compounds that predominantly block the CCK-AR, while spiroglumide and itriglumide glutamic derivatives preferentially antagonize the CCK-BR [28]. The rationale for using proglumide over more highly selective antagonists include the finding that stellate cells and fibroblasts possess both CCK-A and CCK-B receptor types, and inhibition of collagen production from activated stellate cells is reported with blockade of both receptors [29]. Highly potent CCK-A antagonists impair gallbladder emptying [30], which may result in acute cholecystitis. The anti-acid effects of proglumide were initially described by Rovati [31]. Bignamini et al. performed PK studies in rats that demonstrated rapid absorption of proglumide by all three routes of administration (intravenous, intramuscular, and oral) [32] possibly due to its high lipid/water partition coefficient. In human subjects after a single oral dose, they found the Tmax of proglumide occurred after about 1 h and Cmax was about 8000 ng/mL [32]; these values are similar to those found in the subjects from our investigation. These researchers compared their PK results in rats with those of human subjects, and they concluded that the metabolism and kinetics in the rats and humans were comparable and that the recommended oral dose of proglumide in man was 400 mg TID or 1200 mg/day [32]. The steady-state plasma level of the drug during therapeutic dosage regimens was estimated to be in a range around 60% of the peak level of the single administration with terminal half-life of about 24 h [32]. Although specific information about proglumide in those with cirrhosis or hepatic impairment has not previously been studied, extensive PK studies were performed with an analogue of proglumide, dexloxiglumide [33]. Dexloxiglumide ingestion showed no difference between 23 male and female subjects, aged 18–75 years (12 healthy and 12 hepatically impaired; 4 mild, 4 moderate, and 4 severe based on Child-Pugh score), after receiving a single oral dose of dexloxiglumide 200 mg in regard to absorption and excretion.

There are several unique features of proglumide that may favor its use in subjects with liver diseases and liver impairment. The first characteristic is that the drug is orally bioavailable with rapid biodistribution, and the majority is excreted in the urine within 3 h. Another feature includes its mild suppression of gastric acid, similar in potency to histamine-2 receptor antagonists, but it is the only CCK receptor antagonist that does not raise serum gastrin levels [34]. In rodents, proglumide is also the only CCK receptor antagonist that increases bile flow [35] and decreases bile acid concentration. We previously showed in mice fed a NASH-inducing diet [19] that proglumide significantly reduced serum bilirubin levels, suggesting that proglumide was stimulating bile flow and reversing hepatocyte injury. For this reason, the role of proglumide may be beneficial in those with liver diseases.

Proglumide was tested in a phase 1 trial to test the safety and best dose for human subjects with fatty liver disease (NCT04152473). Proglumide is an older drug that was originally developed for peptic ulcer disease [36], but when proton pump inhibitors were developed, commercialization of proglumide ceased in the US. Proglumide is orally bioavailable and was used in peptic ulcer clinical trials at a dose of 1200 mg. Since treatment with proglumide exhibited antifibrotic effects in the liver and decreased the risk for HCC in animal models, proglumide may have potential beneficial effects in subjects with cirrhosis or as an adjuvant therapy in those with HCC. In order to conduct these proposed clinical studies, we sought to test the safety and pharmacokinetics of proglumide in subjects that are hepatically impaired.

## 5. Conclusions

In the current investigation, we demonstrated that the cholecystokinin receptor antagonist proglumide has similar absorption, biodistribution, and clearance in those with Child-Pugh A or B cirrhosis compared with healthy controls. Since the Cmax and Tmax values were comparable, oral dosing adjustments should not be required in those subjects with Child-Pugh A or B cirrhosis with normal renal clearance.

Because of its antifibrotic properties we observed in murine models and its ability to also decrease hepatic steatosis, this agent may be useful in treating subjects with cirrhosis, nonalcoholic steatohepatitis, or alcohol-related liver disorders. Since proglumide monotherapy also decreases the growth of human HCC xenografted to mice and improves pain without worsening hepatic function, the application of proglumide therapy could potentially be extended to those with hepatocellular cancer.

## 6. Patents

Georgetown University has a patent for the use of proglumide in liver disease.

## Figures and Tables

**Table 1:** Patient demographics and baseline laboratory tests.

Characteristic/Test	Value	Healthy Controls	Child-Pugh A	Child-Pugh B	*p*-Value
**Age (years)**	Mean	50.25 ± 3.8	53 ± 8.7	55 ± 2.3	0.869
	Range	39–55	30–72	51–59	
**Gender**	%Male	50	25	100	
	%Female	50	75	0	
**Race**	Caucasian	2	2	2	
	Black	1	2	0	
	Asian	1	0	1	
**BMI**	Mean	28.8 ± 3.6	30.5 ± 3.9	40.1 ± 5.2	0.203
	Range	23.2–39.2	23.5–41.2	29.9–46.7	
**Creatinine (mg/dL)**	Mean ± SEM	0.798 ± 0.069	0.65 ± 0.064	0.88 ± 0.107	0.177
**T. Bilirubin (mg/dL)**	Mean ± SEM	0.37 ± 0.05	0.55 ± 0.12	1.83 ± 0.30 *	0.03 *
**Albumin (g/dL)**	Mean ± SEM	4.45 ± 0.13	4.28 ± 0.11	3.55 ± 0.35	0.0698
**PT (s)**	Mean ± SEM	13.18 ± 0.10	14.85 ± 0.69	15.7 ± 0.97	0.02 *
**MELD**	Mean ± SEM	6.0 ± 0	7.5 ± 0.87	10 ± 1.5	0.043 *

* Represents a significant difference compared to healthy controls

**Table 2:** Comparison of AUC for Cmax of serum proglumide values between healthy controls and Child-Pugh A or B cirrhosis with 90% confidence level.

Comparisons				BE Limit		90% Confidence Interval	
		*p*-Value	Point Estimate	80%	125%	Lower CI	Upper CI
**Child-Pugh A** **Cirrhosis vs. Healthy Controls**	AUC	0.1284	−0.4051616	−0.324129	−0.506452	−0.8703133	0.0599901
	Cmax	0.6809	0.2736093	0.2188875	0.3420116	−1.897749	2.444967
**Child-Pugh B** **Cirrhosis vs. Healthy Controls**	AUC	0.3448	−0.2496918	−0.199753	−0.312115	−0.7760883	0.2767048
	Cmax	0.6474	0.321576	0.2572608	0.40197	−1.467989	2.111141
**Child-Pugh B** **Cirrhosis vs. Child-Pugh A Cirrhosis**	AUC	0.498	0.1554698	0.1243759	0.1943373	−0.3042427	0.6151823
	Cmax	0.9112	0.04796672	0.0383734	0.0599584	−0.8406249	0.9365584

**Table 3:** Laboratory values (chemistry, coagulation, and blood count) obtained prior to proglumide and after ingestion of proglumide in subjects with Child-Pugh A or B cirrhosis.

Blood Test	Child-Pugh A		Child-Pugh B	
	Pretreatment	Post-Treatment	Pretreatment	Post-Treatment
**ALT (units/L)**	31.00 ± 5.90	21 ± 13	26 ± 3	26.67 ± 3.53
**AST (units/L)**	35.25 ± 2.78	25 ± 5	52.5 ± 1.5	36.7 ± 10.5
**T Bili (mg/dL)**	0.55 ± 0.119	0.60 ± 0	1.83 ± 0.15	1.227 ± 0.29
**Albumin (gm/dL)**	4.28 ± 0.111	4.5 ± 0.4	3.55 ± 0.35	3.70 ± 0.379
**Alkaline** **Phosphatase units/L)**	183.5 ± 61.8	208 ± 133	92.5 ± 24.5	89 ± 21.9
**Creatinine (mg/dL)**	0.650 ± 0.064	0.7350 ± 0.045	0.88 ± 0.107	0.930 ± 0.147
**INR**	1.15 ±0.087	1.0 ± 0.0	1.35 ± 0.15	1.3 ± 0.1
**Prothrombin Time (s)**	14.85 ± 0.695	12.3 ± 1.3	15.7 ± 0.97	14.45 ± 0.05
**Platelets (k/µL)**	202.3 ± 62.6	216 ± 25	106 ± 44.5	185 ± 1.6
**Glucose (random) (mg/dL)**	143.8 ± 29.4	137.5 ± 42.5	111 ± 4	93.7 ± 10.2
**Hemoglobin (g/dL)**	11.75 ± 1.15	13.7 ± 3.7	13.95 ± 0.45	13.7 ± 1.0
**White Blood Count (k/µL)**	5.225 ± 0.972	8.3 ± 1.7	5.8 ± 0.1	5.1 ± 1.4

## Data Availability

Data from this clinical trial will be available on www.clinicaltrials.gov, accessed on 20 February 2022, under registration # NCT04814602.

## Supplementary Materials

The following supporting information can be downloaded at: https://www.mdpi.com/article/10.3390/pharmaceutics14030627/s1: Extended methods: Preparation of proglumide Standards and Internal Standard Stock Solutions. Table S1. Liquid chromatography gradient method details. Table S2. UPLC-MRM-based mass spectrometric analysis of proglumide in serum. Table S3. UPLC-MRM-based mass spectrometric analysis of proglumide in urine. Figure S1. UPLC-MRM-based mass spectrometric analysis of proglumide in serum: A sample chromatogram of eluted proglumide and internal standard (4-NBA). Figure S2. UPLC-MRM-based mass spectrometric analysis of proglumide in urine: A sample chromatogram of eluted proglumide and internal standard (4-NBA).
