# Supplementary material for: Safety and Pharmacokinetic Assessment of Oral Proglumide in Those with Hepatic Impairment
Source: Pharmaceutics. 2022 Mar 12;14(3):627. doi: 10.3390/pharmaceutics14030627 (PMC8948956; doi:10.3390/pharmaceutics14030627)
Supplement: Supplementary file 1 [file pharmaceutics-14-00627-s001.zip › pharmaceutics-1626315-supplementary.pdf]

pharmaceutics

Supplementary Materials: Safety and Pharmacokinetic Assessment of Oral Proglumide in Those with Hepatic Impairment

Christine Hsu, Sunil Bansal, Hong Cao, Coleman I. Smith, Aiwu He, Martha D. Gay, Yaoxiang Li, Amrita Cheema and Jill P. Smith

Methods: Preparation of Proglumide Standards and Internal Standard Stock Solutions

(a) Extraction buffer (Internal standard): A 1 mg/mL solution (Stock A) of 4-Nitrobenzoic acid was prepared by dissolving 1 mg of 4-NBA in 1 ml of methanol and vortexed well for 30 seconds. 15 μL of stock-A was added to 100 ml of methanol to make the final nominal concentration of 4-NBA as 150 ng/ml.

(b) Proglumide (standard): A 1 mg/mL solution (Stock B) of proglumide was prepared by dissolving 1 mg of the drug in 1 ml of methanol and vortexed well for 30 seconds. The stock-C (50 μg/ml) was prepared by making up 50 μL of stock-B up to 1 ml in methanol. To generate calibration curve, stock-C was serially diluted in extraction buffer to generate concentrations with control matrices in similar ratios as in actual test samples. Similarly, another set of standards in the similar concentration range was prepared using extraction buffer in blank solvent (methanol) for recovery estimation. Also, 3 QC samples were prepared in blank matrices and solvent, using extraction buffer.

Table S1. Liquid chromatography gradient method.

S. No	Time (min)	Flow rate (ml/min)	Solv A	Solv. B	Curve
1	0	0.2	100	0	6
2	1.0	0.2	100	0	6
3	1.5	0.35	100	0	6
4	3.0	0.5	20	80	6
5	3.5	0.5	20	80	6
6	4.0	0.2	100	0	6
7	5.0	0.2	100	0	6

Table S2. UPLC-MRM based mass spectrometric analysis of proglumide in serum over a range from 5 ng/ml to 10 μg/ml: Calibration curve details.

STD (ng/ml)	RT	Area	IS Area	Response	Conc (ng/ml)	%Dev	S/N
2.5	3.1	4	13770	0	3	17	72
5	3.1	4	9812	0	4	-13	93
10	3.1	6	8197	0	6	-36	126
25	3.1	18	6652	0	21	-14	311
50	3.1	34	5976	1	44	-13	161
100	3.1	73	4802	2	115	15	1582
250	3.1	187	5864	5	240	-4	1240
500	3.1	355	5026	11	530	6	6233
1000	3.1	746	5022	22	1114	11	16111
2500	3.1	1493	4036	55	2774	11	32636
5000	3.1	2708	3642	112	5575	12	50545
7500	3.1	4343	4236	154	7689	3	92907
10000	3.1	5893	5006	177	8827	-12	103230

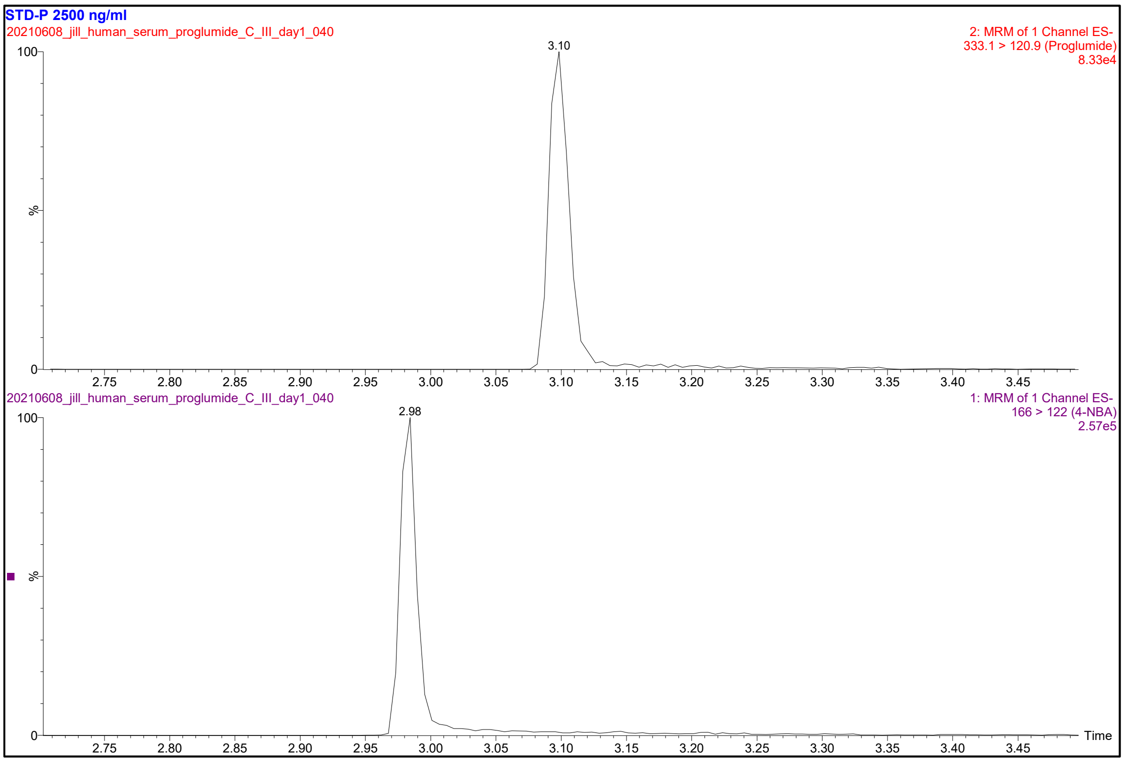

Figure S1. UPLC-MRM based mass spectrometric analysis of proglumide in serum: A sample chromatogram of eluted proglumide and internal standard (4-NBA).

Table S3. UPLC-MRM based mass spectrometric analysis of proglumide in urine over a range from 2.5 ng/ml to 100 µg/ml: Calibration curve details.

STD (ng/ml)	RT	Area	IS Area	Response	Conc (ng/ml)	%Dev	S/N
5	3.1	8	14186	0			15
10	3.09	14	14588	0	15	53	57
25	3.09	24	15221	0	35	39	61
50	3.1	42	15254	0	76	53	270
100	3.1	102	15580	1	207	107	2450
250	3.1	241	16427	2	490	96	1283
500	3.09	476	16363	4	987	98	1633
1000	3.1	846	17127	7	1690	69	4627
2500	3.1	2104	17668	18	4102	64	6078
5000	3.1	3978	17071	35	8045	61	3826
10000	3.1	6505	16672	59	13484	35	38413
25000	3.1	12448	16699	112	25778	3	14533
50000	3.1	22009	15563	212	48921	-2	29608
75000	3.1	29564	14188	313	72090	-4	24094
100000	3.1	36336	12565	434	100059	0	131501

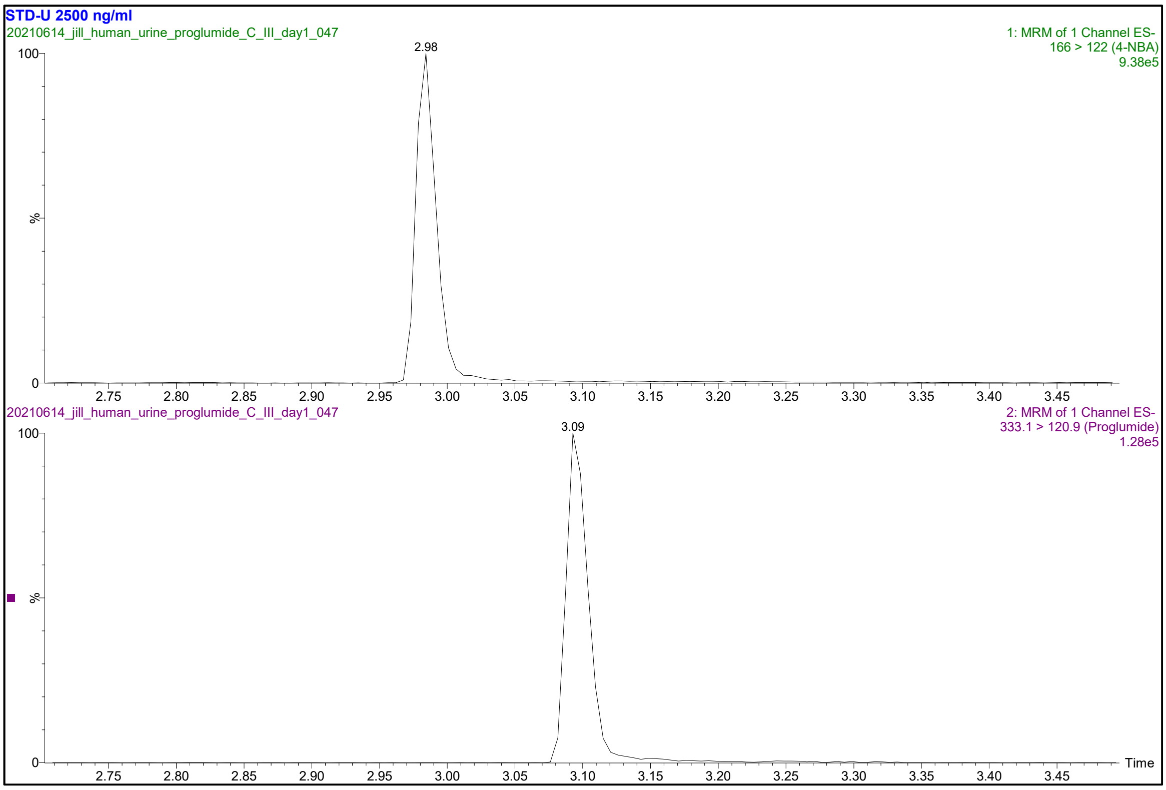

Figure S2. UPLC-MRM based mass spectrometric analysis of proglumide in urine: A sample chromatogram of eluted proglumide and internal standard (4-NBA).
